# Predicting individual dry matter intake in Holstein × Gyr cows using behavior-monitoring sensor, phenotypic, and weather data with supervised machine learning

**DOI:** 10.3168/jdsc.2025-0850

**Published:** 2026-01-16

**Authors:** Camila S. da Silva, Tadeu E. da Silva, Anna L.L. Sguizatto, Andreia F. Machado, Abias S. Silva, João H.C. Costa, Mariana M. Campos, Domingos S.C. Paciullo, Carlos A.M. Gomide, Mirton J.F. Morenz

**Affiliations:** 1 Brazilian Agricultural Research Company, Embrapa Mid-North, Teresina, PI 64006-220, Brazil; 2 University of Vermont, Department of Animal and Veterinary Sciences, Burlington, VT 05405; 3 Brazilian Agricultural Research Company, Embrapa Dairy Cattle, Juiz de Fora, MG 36038-330, Brazil; 4 Brazilian Agricultural Research Company, Embrapa Western Amazon, Manaus, AM 69010-970, Brazil

## Abstract

Accurate estimation of DMI is essential for optimizing nutrition, efficiency, and economic performance in modern dairy herds. However, most existing equations to estimate DMI are designed for herd-level predictions in purebred Holstein cows. This study evaluated the accuracy and precision of machine learning (ML) algorithms to predict daily individual DMI in Holstein × Gyr crossbred lactating cows using a supervised and integrative approach that combined behavior monitoring data, cow phenotypes, and weather features. Data from 31 cows were individually and consecutively collected over 18 d. Twenty-two cows (71% of the dataset) were used to train 4 linear regression models (multiple linear, ridge, lasso, and elastic net) and 3 ensemble algorithms (random forest, gradient boosting, and extreme gradient boosting) through leave-one-group-out cross-validation, with the number of folds equal to the number of cows (*k* = 22). The remaining 9 cows were used as an external test set. Among all algorithms, Gradient boosting achieved the best overall performance, yielding moderate precision (R^2^ = 0.68) and accuracy (root mean squared error = 1.60 kg/d) metrics on test data. Our results indicate that gradient boosting is more suitable for capturing complex nonlinear relationships underlying daily DMI compared with the other models evaluated. Further advancements in ML-based DMI prediction should consider integrating intra- and interindividual variability in feeding behavior and accounting for animal-specific effects.

## Highlights

• Machine learning was applied to predict daily DMI in Holstein × Gyr cows.
• Phenotype, behavior, and weather data were integrated into ML models.
• GB showed the best predictive accuracy and precision.
• Body weight and milk yield were the largest contributors to predicted DMI values.
• Inter- and intra-animal effects could improve future ML-based predictions.

## Graphical Abstract

**Figure:**
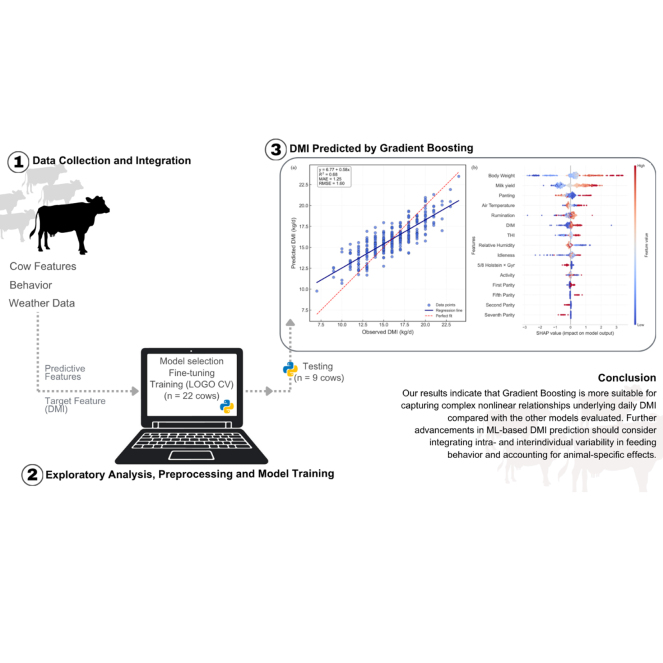
**Summary:** Individual dry matter intake (DMI) of Holstein × Gyr lactating cows was predicted via supervised machine learning (ML) using a data-integrative approach. In step 1, cow features, behavior, weather data, and individual DMI were continuously collected from 31 cows over 18 days, yielding 558 observations; in step 2, predictor and target (DMI) features were integrated into supervised ML models, which were fine-tuned and trained using data from 22 cows via leave-one-group-out cross validation (LOGO CV). The best model, gradient boosting (GB), was then selected based on the models' performance metrics on test data (n = 9 cows; step 3). Among all regression models evaluated, GB showed the best predictive ability on unseen cows for individual prediction of DMI. Body weight and milk yield were the most influential contributors to prediction. MAE = mean absolute error; RMSE = root mean squared error.

Feed costs have long represented the largest expense in dairy cattle operations. As such, both short- and long-term management decisions, such as dry-off, culling, feeding strategies, genetic selection, and monitoring of feed income over cost are often driven by feed intake (Huzzey et al., 2007; Toghiani et al., 2024). Nonetheless, most individual DMI reported in the literature data have been collected in research settings. This is primarily due to the challenge of measuring DMI on-farm in group-feeding systems and the need for specialized infrastructure to track both feed offered and refused on a per-animal basis (Brown et al., 2022).

Given these challenges, several mathematical equations have been developed to estimate DMI. The most up-to-date and widely accepted models are 2 empirical regression equations (NASEM, 2021): the first considers only animal-related traits, such as parity, milk energy output, BW, BCS, and DIM, whereas the second integrates these traits with dietary features to enhance prediction accuracy and precision. Both models are particularly suited for purebred dairy cows raised under temperate climates. In tropical regions such as Brazil, where feed composition, genetic composition, and environmental conditions differ substantially from those in temperate climates, another system of equations for DMI prediction in crossbred and purebred Holsteins was recently proposed (BR-Leite; Oliveira et al., 2024). However, all these models are inherently limited in their ability to accurately predict individual DMI because they are built from a wide range of characteristics to predict the DMI for a given group of cows. In contrast, advances in artificial intelligence have created opportunities to develop machine learning (**ML**) models that integrate routinely collected herd- and cow-level data to generate individual predictions of DMI and learn intake patterns from large datasets without relying on assumptions about data distribution or constrained mathematical structures (Ding et al., 2022). Due to these advantages, ML algorithms have attracted increasing attention for predicting DMI in both beef and dairy cattle systems (Salleh et al., 2023; ArunKumar et al., 2025).

In a study comparing artificial neural networks and stacked ensembles with multiple linear regression and partial least squares (**PLS**) regression across 4 feature sets, Martin et al. (2021) reported slightly higher precision and lower prediction errors for the linear models, suggesting that linear regression approaches may be sufficient for predicting DMI in mid-lactation Holstein cows. These findings were later corroborated by Salleh et al. (2023), who evaluated the performance of PLS, support vector machines, and random forests (**RF**) for individual DMI predictions across subsets of DIM. Conversely, artificial neural networks achieved the best DMI predictions in lactating Holsteins compared with PLS across 5 feature sets (Dórea et al., 2018), highlighting the importance of exploring ML techniques under different prediction contexts. For instance, Holstein × Gyr crossbred lactating cows may differ considerably from purebred Holsteins in behavior, intake level, and performance (Angelo et al., 2022; Quirino et al., 2022), which could markedly affect predicted DMI. Therefore, the objective of the present study was to predict, at the individual cow level, the daily DMI of lactating Holstein × Gyr crossbred cows using an integrative approach that combined behavior-monitoring collar (**BMC**), phenotypic, and weather data as input features in supervised ML algorithms. We hypothesized that such an approach would allow accurate and precise individual predictions of DMI in lactating crossbred cows, providing valuable resources to enhance nutritional management of dairy production systems.

All animal procedures were approved by the Embrapa Dairy Cattle Animal Care and Use Committee (Juiz de Fora, Minas Gerais, Brazil; Protocol CEUA-EGL 2265141022). Thirty-seven Holstein × Gyr (Girolando) cows with mean ± SD DIM of 194 ± 50, 536 ± 56 kg of BW, and 15.6 ± 2.3 kg/d of MY were evaluated. The cows exhibited different breed compositions within the Girolando breed (22.6% 1/2 Holstein × Gyr, 54.8% 3/4 Holstein × Gyr, and 22.6% 5/8 Holstein × Gyr). The experiment was initially designed for grazing cows, and the sample size (n  =  37) was defined based on previous pasture-based DMI prediction studies (Oudshoorn et al., 2013; Rombach et al., 2019). Due to limited animal and device availability, this sample size was maintained when the trial was conducted in confinement.

The cows were sourced from the Girolando herd of Embrapa Dairy Cattle (Coronel Pacheco, MG, Brazil). To facilitate management and reduce health issues during the experiment, they were screened for health and adaptability to freestall housing and treated for ectoparasites 7 d before transfer to the experimental site. The cows were also fitted with a BMC (CowMed, Chip Inside LTDA, Santa Maria, RS, Brazil) that tracked real-time rumination, activity, idleness, and panting every 15 min (Lovatti et al., 2024). The collar was positioned on the left side of the neck, following the manufacturer's instructions.

The trial was conducted at the Multi-User Laboratory of Bioefficiency and Livestock Sustainability (Embrapa Dairy Cattle, Coronel Pacheco, MG, Brazil) in a freestall barn equipped with ventilation and sprinklers. No group allocation was performed in this study, as no specific treatments were tested. Upon arrival, the cows were weighed and assigned to 2 barn modules containing automatic feeders for real-time measurement of individual feed intake (AF-1000, Ponta Agro Ltd., Betim, MG, Brazil; Chizzotti et al., 2015), shared water troughs, and individual stalls with rubber mattresses and wood shavings. A meteorological station (Ponta Agro Ltd., Betim, MG, Brazil) installed in the central area of the barn recorded relative air humidity (**RH**) and dry bulb air temperature (**Temp**) during the experiment. Air temperature and RH were used to calculate the temperature-humidity index (**THI**; NRC, 1971), where THI = (1.8 × Temp + 32) − (0.55 − 0.0055 × RH) × (1.8 × Temp − 26). The Temp, RH, and THI during the study were 24.0°C, 80.2% and 73.3, respectively (Table 1).

**Table 1:** Descriptive analysis of cow and behavioral features from Holstein × Gyr crossbred lactating cows (n = 31) used in this study^1^

Variable	Mean	Minimum	Q1	Median	Q3	Maximum	SD
Cow features							
Mean BW, kg	553.05	434.50	517.50	542.00	601.00	688.00	59.92
DMI, kg/d	16.17	6.95	14.04	16.22	18.21	26.31	3.05
DMI, % BW	2.94	1.15	2.60	2.95	3.29	4.35	0.52
Milk yield, kg/d	12.44	6.50	10.80	12.30	14.00	19.60	2.19
ECMY, Mcal/d	14.40	5.51	12.53	14.25	16.28	22.30	2.85
FCMY, kg/d	15.82	6.18	13.69	15.58	18.03	24.30	3.13
Behavior features							
Rumination, min/d	410.21	112.75	336.70	439.75	494.32	600.71	107.56
Panting, min/d	249.85	122.04	171.26	196.27	275.18	753.63	124.41
Activity, min/d	161.47	30.67	120.06	160.42	204.54	343.00	60.70
Idleness, min/d	618.47	432.29	564.46	602.63	658.97	877.67	81.72
Weather features							
Relative air humidity, %	80.20	71.69	78.28	79.68	81.57	88.51	3.65
Air temperature, °C	23.99	20.70	23.66	24.13	25.09	26.58	1.52
THI	73.31	68.00	72.65	73.36	75.29	77.02	2.44

1 Q1 = first quartile; Q3 = third quartile; ECMY = energy-corrected milk yield, calculated according to the equation proposed by NASEM (2021); FCMY = milk yield corrected for 3.5% fat (Sklan et al., 1992); THI = temperature-humidity index (NRC, 1971).

The trial included a 15-d adaptation period to the facilities followed by 18 d of uninterrupted data collection. Throughout the experiment, cows were fed a TMR (70:30 forage-to-concentrate ratio) formulated according to NRC (2001) recommendations for cows producing 15 kg/d of milk with 3.5% milk fat content. The TMR consisted of corn silage and a concentrate mix (ground corn, soybean meal, feed-grade urea, and a mineral supplement) fed once daily (0730 h).

Milk yield was recorded twice daily, at 0800 and 1400 h, using automated meters integrated into the milking system (HB30, DeLaval Brasil, Jaguariúna, SP, Brazil). Individual milk samples were collected at 4 consecutive milkings in each of the last 2 wk of the data collection period for composition analysis (Master Complete AK511, AKSO Produtos Eletrônicos Ltda., São Leopoldo, RS, Brazil) and estimation of fat- and energy-corrected milk yield according to Sklan et al. (1992) and NASEM (2021; Table 1).

Cows were weighed again on the final day of the experiment, and their ADG was calculated as the difference between final and initial BW divided by the number of experimental days. The ADG was then used to estimate daily BW. Behavioral data obtained from the BMC, DMI, MY, and animal traits (breed composition, parity, and daily BW) were combined for preprocessing and model development. Six cows were completely removed from the original dataset due to faulty data collection by the BMC, automatic feeders, or both, leading to excessive missing data. Other isolated missing values (0.26%) were imputed using cow-specific means of the corresponding variable (e.g., DMI, MY, rumination). The final dataset comprised 31 cows and 558 observations (Table 1).

The collected data were preprocessed and analyzed using the Python programming language (Anaconda Software Distribution, version 2.6.3, Anaconda Inc.). All data collection, outcome assessment, and data analysis were conducted by the same researcher, who was responsible for the entire experimental procedure. Categorical variables (e.g., breed composition and parity) were one-hot encoded. The predictor variables were assessed for collinearity using Spearman correlation coefficients. This analysis was also used to examine the relationships between predictors and the target variable (DMI). Highly collinear variables (|r| > 0.70) that showed weak associations with DMI, such as ECM yield, FCM yield, and THI, were excluded from preselected linear models. Conversely, all predictors were retained in preselected ensemble algorithms, given their lower sensitivity to collinearity and greater ability to effectively handle correlated features (Sigrist, 2022). Finally, all predictors were standardized to have a mean of 0 and SD of 1 using the StandardScaler class of the preprocessing module within Python's Scikit-Learn library (Scikit-Learn Developers, 2025).

Before any modeling steps were performed, the full dataset was randomly divided into a training set (22 cows; ∼71%) and a test set (9 cows; ∼29%). Model development and tuning were conducted exclusively within the training set. The final model was then validated using the fully independent test set of 9 cows. Seven multiple regression algorithms were initially selected for model training: multiple linear regression (**MLR**), ridge regression (**RR**), lasso regression (**LA**), elastic net (**EN**), RF, gradient boosting (**GB**), and extreme gradient boosting (**XGBoost**). Assumptions for parametric models were assessed using standard diagnostic plots and Spearman's correlation. The ensemble algorithms are nonparametric models, and thus exempt from strict assumptions about data distribution. Model training was performed via leave-one-group-out cross-validation (**LOGO CV**) implemented in the Scikit-Learn's *model_selection* module. In LOGO CV, each cow was defined as a group of observations, resulting in 22 folds (*k*). At each iteration, data from one cow were completely excluded from the training set and used solely for validation, whereas the remaining cows (*k* − 1) composed the training set. This strategy is particularly appropriate for datasets with grouped or hierarchical structures (Coelho Ribeiro et al., 2021), as it prevents data leakage between individuals and provides a robust assessment of the model's ability to generalize to unseen subjects.

Except for MLR, the hyperparameters of all models were optimized using a grid search approach combined with the LOGO CV described above. Hyperparameter tuning was performed using Scikit-Learn's *GridSearchCV* function, with the mean squared error (**MSE**) used as the scoring metric. For RR and LA, 50 equally spaced values of the regularization parameter (α) ranging from 0.01 to 250 were evaluated. The EN model was evaluated under the same α grid as RR and LA, with an additional search over the *l1_ratio* values (0.010, 0.025, 0.050, 0.075, 1.00). For the RF model, the combinations tested included the number of trees (100, 200, 300), maximum tree depth (none, 5, 10, 15), minimum number of samples per leaf (1, 2, 4), and number of features considered at each split ('*sqrt*'). The GB model was tuned for the number of estimators (100, 200, 300), learning rate (0.01, 0.05, 0.1), maximum depth (3, 5, 7), minimum samples per leaf (1, 2, 4), subsampling fraction (0.7, 0.85, 1.0), and number of features considered at each split ('*sqrt*'). Finally, the XGBoost model was optimized for the number of estimators (100, 200, 300), learning rate (0.01, 0.05, 0.1), maximum depth (3, 5, 7), subsampling fraction (0.7, 0.85, 1.0), column subsampling rate (0.7, 0.85, 1.0), L1 regularization term (*reg_alpha*: 0, 0.1, 0.5, 1), L2 regularization term (*reg_lambda*: 0.5, 1, 2, 5), and minimum child weight (1, 3, 5).

After hyperparameter optimization, the final models were retrained and evaluated using the LOGO CV procedure with the best parameter settings to estimate validation performance metrics, including mean absolute error (**MAE**; kg/d), mean absolute percentage error (**MAPE**; %), MSE (kg^2^/d^2^), root mean squared error (**RMSE**; kg/d), and R^2^ (Table 2). The optimized models were further tested for DMI prediction using the independent set (n = 9 cows). Among all algorithms, GB showed the best overall performance on unseen cows and was therefore selected as the final model. The relative importance of each predictor was assessed by Shapley additive explanations (**SHAP**) with the *TreeExplainer* method within the *shap* library. By employing the concept of cooperative game theory, SHAP analysis assigns a Shapley value to each feature and quantify their individual contribution to the model's predictions, thereby improving interpretability (ArunKumar et al., 2025).

**Table 2:** Performance of preselected regression models for predicting daily DMI in lactating Holstein × Gyr cows

Model	Performance metrics^1^				
	MAE	MSE	MAPE	RMSE	R^2^
Multiple linear regression	1.792	5.301	0.119	2.302	0.346
Ridge regression	1.860	5.657	0.124	2.378	0.302
Lasso regression	1.792	5.293	0.119	2.301	0.347
Elastic net	1.792	5.296	0.119	2.301	0.347
Random forest	1.589	4.102	0.107	2.025	0.494
Gradient boosting	1.248	2.570	0.083	1.603	0.683
Extreme gradient boosting	1.691	4.699	0.113	2.168	0.420

1 MAE = mean absolute error (kg/d); MSE = mean squared error (kg^2^/d^2^); MAPE = mean absolute percentage error (%); RMSE = root mean squared error (kg/d). Performance metrics represent the quality of predictions of optimized models on an independent dataset (9 cows and 18 data points per cow).

The MAE, MSE, MAPE, RMSE, and R^2^ values for predictions of DMI by the GB model were 1.25 kg/d, 2.57 kg/d^2^, 8.3%, 1.60 kg/d, and 0.68 (Table 2), respectively. Residual analysis showed that prediction errors were mostly random and nonsystematic. However, a slight tendency toward underestimation was observed at higher predicted DMI values, suggesting that the GB model had reduced predictive accuracy for cows with high DMI.

Our SHAP analysis revealed that BW and total MY were the most influential features in the model output, with higher BW and MY values associated with higher predicted DMI (Figure 1b). Panting, Temp, and rumination followed as the next most influential features. However, based on distribution of their SHAP values, it is not possible to infer that higher levels of panting, rumination, and air temperature increased or decreased predictions of DMI, possibly due to nonlinear relationships between these features.

**Figure 1:**
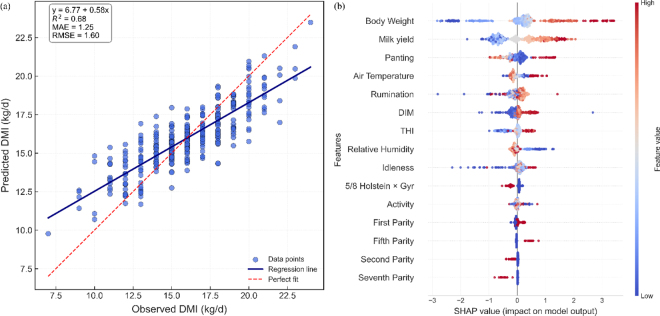
Observed DMI versus DMI predicted (kg/d) in lactating Holstein × Gyr cows (a) and Shapley additive explanations (SHAP) values of predictors on model output (b). Displayed predictions on (a) were generated using the gradient boosting model fitted with all predictor features. Each data point represents one observation. The dashed red line corresponds to y = x, indicating perfect agreement between predicted and observed values. The solid dark blue line represents the linear regression fitted to the data, showing the actual prediction trend. Model bias (MAE, kg/d), R^2^, root mean squared error (RMSE), and intercept (β_0_) and slope (β_1_) coefficients are shown in the upper left corner. The SHAP values (b) are shown for the 15 most important predictive features.

Our performance metrics are in line with previous studies that have applied sensor-based data and ML algorithms to predict DMI, reporting a wide range of predictive performance (R^2^ = 0.07–0.82; RMSE = 1.59–3.42), depending on the predictors and datasets used (Martin et al., 2021; Brown et al., 2022; Shadpour et al., 2022; Siberski-Cooper et al., 2022; Salleh et al., 2023). Consistent with maintenance and production nutritional requirements—the main drivers of feed intake (NRC, 2001)—our final model confirmed the well-established influence of BW and MY on DMI. However, model fit may have been influenced by biological variability in behavioral patterns, limitations in device accuracy and precision, or a combination of these factors, as rumination and panting showed the largest deviations from the mean in our dataset (Table 1) and were ranked among the most influential features.

It is critical to acknowledge that wearable sensors provide estimations of behavioral times rather than direct measurements. As emphasized by Siberski-Cooper et al. (2022), algorithms used in commercial sensors are optimized to detect specific events and adjusted to a series of internal thresholds, which may introduce noise into downstream modeling efforts. Last, Liang et al. (2021) reported a moderate correlation (r = 0.47) between DMI and intake during the first 2 h after feed provision, in contrast to the daily DMI data used in the present research. This suggests that targeting specific postfeeding windows may improve model performance in future DMI prediction studies using integrative approaches.

It should be noted that the nonbehavioral predictors included in our model were selected for their established or potential associations with DMI. For instance, DMI was strongly and negatively correlated with THI (r = −0.82) in a meta-analysis by Chang-Fung-Martel et al. (2021), reflecting the impact of heat stress on feed intake. In contrast, MY is known to positively influence DMI (Hristov et al., 2000), given its role as a major driver of energy demand (NRC, 2001). Martin et al. (2021) demonstrated that combining MY and composition, metabolic BW, and sensor-derived behavioral data provided the most accurate and precise DMI predictions (RMSE = 1.68; R^2^ = 0.80) across 4 predictive algorithms. In the present study, 7 regression algorithms were evaluated, and GB achieved the best overall performance. Model accuracy and precision could potentially be improved with a larger dataset, inclusion of more animals, a refined set of predictors, and integration with random animal variability associated with genetic background and BW composite, which largely affect predicted DMI (Toghiani et al., 2024). Although such variance is typically accommodated by general linear mixed models, GB and the other 6 algorithms evaluated in this study do not inherently account for random effects. Combining boosting frameworks with mixed-model structures, for instance, through Gaussian process boosting regression (Sigrist, 2022), may enhance the precision and accuracy of individual DMI predictions in future studies of cow-level predictions of DMI in Holstein × Gyr lactating cows.

Although our findings provide valuable insights for future model improvement, certain limitations of this analysis should be acknowledged. First, DMI was estimated using electronic feeders rather than direct weighing of feed offered and orts. According to the manufacturer, a deviation of up to 5% from manual DMI measurements can be expected. To minimize this discrepancy, the feeding system was calibrated weekly, whereas calibration is typically performed only when anomalous values are detected under commercial settings. Second, daily BW was estimated based on initial and final measurements rather than direct daily records. Consequently, deviations from true BW may have occurred; however, daily weighing would be impractical under field conditions. Third, model development relied solely on features commonly available on-farm and was based on a small sample size. Therefore, as discussed previously, we cannot rule out the possibility that a larger dataset and the inclusion of additional features, especially cow-related ones, could improve predictions further. Last, substantial day-to-day variation in DMI and behavioral features were observed within individual cows, but the effect of such intra-animal and interdevice variability on prediction performance remains underreported and could not be measured in the current study.

Collectively, our results indicate that GB can achieve moderate precision and accuracy for individual-level DMI predictions when phenotypic, performance, and weather data are available. Future research should aim to improve sensor reliability, expand dataset size, and investigate behavioral and physiological variability—particularly during postfeeding periods—to support the development of more robust and interpretable DMI predictive models for dairy cattle nutrition.
